# Novel genetic features associated with the recently emerged MDR clade of *Salmonella* Dublin linked to human clinical cases

**DOI:** 10.1128/spectrum.01336-25

**Published:** 2025-07-18

**Authors:** Linghuan Yang, Loredana d‘ Ovidio, Ruixi Chen, Cristina Resendiz-Moctezuma, Chenhao Qian, Martin Wiedmann, Renato H. Orsi

**Affiliations:** 1 Department of Food Science, Cornell University 5922, Ithaca, New York, USA; 2 Food Research Center, Department of Food and Experimental Nutrition, Faculty of Pharmaceutical Sciences, University of São Paulo 28133, São Paulo, Brazil; University of Maryland at College Park, College Park, Maryland, USA

**Keywords:** *Salmonella*, serovar Dublin, comparative genomics, multidrug resistance, oxidative stress, transposon, plasmid, virulence, human, cattle

## Abstract

*Salmonella* Dublin is an enteric pathogen that has adapted to cattle as its primary host. It rarely causes foodborne outbreaks but is frequently associated with invasive and severe infections in humans. However, the underlying mechanisms responsible for its enhanced virulence in humans remain unknown. In this study, we conducted comparative genomic analyses and an oxidative stress assay focusing on *Salmonella* Dublin isolates classified, in a previous study, as human-associated (HA) or non-human-associated (NHA). We found that 82.5% and 20% of the isolates in the NHA group harbor premature stop codons (PMSCs) in *pgtC* and *sadA*, respectively, with disrupted *sadA* possibly contributing to attenuated virulence in these isolates. PMSCs in *yahO* and *ratB* were found in 100% of isolates in the HA group and in 62.5% and 60% of isolates in the NHA group, respectively. In addition, 100% and 42.5% of isolates in the HA group harbor PMSCs in *gsiB* and *zirT*, respectively, with disrupted *zirT* possibly contributing to an enhanced virulence in these isolates. Moreover, a multidrug resistance plasmid (pVPS18S0911-1-like) was strongly associated with HA isolates, which may allow these isolates to cause prolonged infections leading to increased clinical severity. While an IS*1* transposon inserted within *panE*, located between two genes putatively involved in oxidative stress, was observed exclusively among HA isolates, these isolates did not show a significantly different oxidative stress survival to H_2_O_2_ as compared to NHA isolates. Finally, the presence of the *Salmonella* virulence plasmid and the Vi antigen was not associated with the HA isolates.

**IMPORTANCE:**

*Salmonella* Dublin causes a severe extra-intestinal infection in 64% of human cases with a 3% fatality rate. In this study, we compared the genomes of human-associated (HA) *Salmonella* Dublin isolates to non-human-associated (NHA) isolates and identified genetic features associated with the HA or NHA groups, such as single nucleotide polymorphisms in genes putatively involved in virulence, presence of antimicrobial resistance genes in an HA-associated plasmid, and an insertion element between two genes putatively involved in oxidative stress. HA and NHA isolates showed, however, no differences in an oxidative stress survival assay. Overall, this study identified novel genetic features putatively involved in the virulence diversity within *Salmonella* Dublin throughout a combination of phylogenetic analysis, genome-wide association study, and phenotypic assessment, providing insights for future studies to elucidate the mechanisms leading to the severe human infections typically caused by *Salmonella* Dublin.

## INTRODUCTION

*Salmonella enterica* subspecies *enterica* serovar (*S*.) Dublin is one of the most common serovars collected from dairy-beef production in the USA (1, 2). In 2014, according to a National Animal Health Monitoring System study, *S*. Dublin was estimated to be present in 8% of dairy farms in the USA (3). While predominantly host-adapted to cattle, *S*. Dublin has also been reported to infect sheep, horses, swine, and other animals (2, 4, 5).

Unlike other non-typhoidal *Salmonella* serovars, which are mainly associated with gastrointestinal illnesses, *S*. Dublin infection can not only lead to typhoidal disease in cattle, but also result in invasive salmonellosis in humans (6, 7). Calves are especially susceptible to infections caused by this serovar, which may lead to respiratory or bloodstream diseases and even death (8). Conversely, adult cattle infected with *S*. Dublin may remain asymptomatic carriers (8). In humans, fever, osteomyelitis, meningitis, bacteremia, and abdominal cramping can be elicited (9, 10). Although studies have identified the Vi antigen in *S*. Dublin (11, 12), which had only been reported in *Salmonella enterica* serovar Typhi and *Salmonella enterica* serovar Paratyphi C previously (13, 14), it is not clear if the Vi antigen is associated with enhanced human virulence in *S*. Dublin. Another well-studied virulence determinant in *Salmonella* is the *Salmonella* virulence plasmid (pSV), which has only been reported in a few serovars of the subspecies *enterica*, including most isolates of *S*. Dublin. Despite these findings, it remains unclear which virulence factors play a major role in the hypervirulence of certain *S*. Dublin strains.

Chen et al. (15) previously identified a major clade within *S*. Dublin (referred to as Dublin 2-3 in their study) corresponding to 66% of all *S*. Dublin isolates in the National Center for Biotechnology Information (NCBI) Pathogen Detection database. Among the 2,933 isolates within Dublin 2-3, 42% were human isolates and 54% were cattle or cattle-associated (e.g., cattle farm environment) isolates. Based on the prevalence of human isolates, in this study, Dublin 2-3 could be further divided into two groups: the non-human-associated (NHA) group (containing 12% human and 79% cattle-associated isolates), and the human-associated (HA) group (containing 45% human and 52% cattle-associated isolates). To further investigate the genetic mechanisms leading to a potential enhanced human virulence of *S*. Dublin in the HA group, we conducted a detailed analysis of the Dublin 2-3 clade using comparative genomic approaches to identify (i) genetic characteristics, including plasmids, phage-borne genes, and premature stop codons (PMSCs), that are strongly associated with the HA or NHA groups, and (ii) multidrug resistance differences between two groups. In addition, we conducted a phenotypic assay to identify differential oxidative stress survival between the HA and NHA groups.

## MATERIALS AND METHODS

### Isolate selection, whole-genome sequence assembly, tree reconstruction, and genome annotation

Within *S*. Dublin, a clade (Dublin 2-3) containing 66% of all *S*. Dublin isolates has been identified, with previous estimates suggesting its emergence around 1884 (15). This clade includes several multidrug resistance (MDR) isolates, including a number of isolates obtained in the USA after 2012. Here, we identified a recently emerged clonal group with an increased proportion of human isolates within Dublin 2-3. We designated this group as the HA group, while the remaining isolates within Dublin 2-3 (predominantly enriched for cattle isolates with few human isolates) were designated as the NHA group (15). Forty representative isolates were randomly selected from each group for inclusion in the comparative genomic analysis. When available, genome assemblies of the representative isolates were downloaded from NCBI Pathogen Detection (PD) (https://www.ncbi.nlm.nih.gov/pathogens/). For isolates lacking genome assemblies on NCBI PD, raw reads were acquired from the NCBI Sequence Read Archive (https://www.ncbi.nlm.nih.gov/sra/) using fastq-dump (16) and subsequently assembled *de novo* with SKESA (17). QUAST (18) was used to check the quality of all genome assemblies. The assembly quality criteria for inclusion of an assembly in the study included (i) a genome size between 4.0 and 6.0 Mbp, (ii) N50 >20,000 bp, and (iii) <500 contigs. kSNP4 (19) was used to identify core single nucleotide polymorphisms (SNPs) from a total of 81 genomes, including 80 representatives from the HA and NHA groups (see Table S1 for full list), and a *Salmonella enterica* subsp. *enterica* serovar Enteritidis genome (GCF_000623455.2), which was used as an outgroup. Based on the core SNP matrix, a maximum-likelihood tree was then reconstructed using FastTree (20) with the GTR-CAT model and 1,000 bootstrap replicates. Prokka (21) was utilized to annotate the genomes of all representative isolates.

### Identification of genes overrepresented among the HA or NHA group

Panaroo (22), with a default sequence identity of 98%, was utilized to estimate the pan-genome composition among all 80 representative isolates from the HA and NHA groups. Scoary (23) was used to conduct a pan-genome-wide association study (pan-GWAS) to identify genes overrepresented among isolates in the HA or NHA group (hereafter referred to as HA- and NHA-associated genes, respectively), and false discovery rate (FDR) correction was applied to account for multiple comparisons while assessing statistical significance (24). The HA- and NHA-associated genes identified through pan-GWAS (FDR <0.05) were further annotated using InterProScan (25) to provide additional functional insights, especially for those genes initially annotated by Prokka (21) as encoding hypothetical proteins. The lists of HA- and NHA-associated genes were further assessed to identify gene functions that could potentially be involved in *S*. Dublin virulence.

### Identification of mobile genetic elements and putative virulence genes

For each genome assembly, *in silico* identification of putative prophage and plasmid regions was performed using PHASTER (26) and Platon (27), respectively. The HA- and NHA-associated genes identified were classified as phage-borne, plasmid-borne, or chromosomal by assessing whether their coordinates (i.e., contig accession, and start and end positions within a contig as annotated by Prokka) overlapped with the coordinates of plasmids and prophages as identified by Platon and PHASTER, respectively. If a group of HA- or NHA-associated genes was located adjacent to each other, the corresponding DNA sequence encompassing all the HA- or NHA-associated genes within the region was extracted and aligned against the core nucleotide database using BLASTN from the NCBI BLAST website (28). We used cutoffs of 90% sequence identity and 90% query coverage to identify plasmids, phages, or other mobile genetic elements overrepresented among the HA or NHA group. To identify putative virulence factors among HA- and NHA-associated genes, these genes were searched against three virulence databases (PATRIC [29], Victors [30], and VFDB [31]) using BLAST+ (32) with cutoffs of >90% sequence identity and >90% query coverage. All 80 genomes were also searched against well-studied virulence factors including pSV and the Vi antigen. The pSV nucleotide sequence (accession no. NC_010422.1) and Vi antigen amino acid sequence (accession no. WP_001210944.1) were downloaded from the NCBI GenBank and used to create nucleotide and protein BLAST databases, which were searched using the *S.* Dublin genome assemblies analyzed here. Assemblies with matches to either sequence with >90% sequence identity and >90% query coverage were considered as carrying these elements.

### Identification of core SNPs leading to PMSCs overrepresented among the HA or NHA group

Reference-based SNP variant calling was performed to identify core SNPs among the 80 representative *S*. Dublin genomes using kSNP4 (19) with the -vcf flag enabled. All 35 *S*. Dublin genomes (see Table S2 for full list) containing less than five contigs were downloaded from NCBI PD and designated as reference genomes for annotating core SNPs. Based on the annotation, core SNPs resulting in PMSCs were identified, and a pan-GWAS analysis using the program Scoary was performed to identify PMSCs overrepresented in the HA or NHA group. FDR correction was applied to account for multiple comparisons while assessing statistical significance (24).

### *In silico* analysis of antimicrobial resistance (AMR**)**

The methodology described in Chen et al. (15) was used to identify AMR elements in all 80 genomes. Briefly, for each isolate, the presence/absence data for curated AMR elements (point mutations or genes) from the Pathogen Detection Reference Gene Catalog (https://www.ncbi.nlm.nih.gov/pathogens/refgene) were retrieved from the metadata. These distinct AMR determinants were then mapped to one or more drug classes according to the Comprehensive Antibiotic Resistance Database (CARD; [33]). For each isolate, the number of drug classes to which resistance was likely conferred was determined based on the AMR determinants identified in the genome. Isolates harboring AMR determinants conferring resistance to at least three CARD drug classes were classified as putative MDR isolates. Fisher’s exact tests were used to determine whether specific AMR classes were associated with the HA and NHA groups. FDR was used to adjust the individual *P*-values due to multiple testing. In addition, a *t*-test was used to assess whether HA isolates are resistant to a significantly higher number of AMR classes as compared to NHA isolates.

### *Salmonella* Dublin isolates used for hydrogen peroxide killing assay

To assess the functional impact of an HA-specific IS*1* insertion into *panE*, which is located between two genes putatively involved in oxidative stress, we identified eight isolates representing the HA (*n* = 4) and NHA (*n* = 4) groups. These eight isolates were used to conduct a hydrogen peroxide killing assay. The eight isolates were whole-genome sequenced to confirm that the four HA isolates carried the IS*1* transposon within *panE*, while the four NHA isolates carried a full-length copy of *panE* without the IS*1* transposon insertion. Within each group (HA and NHA), two isolates were originally collected from human hosts and two from cattle hosts (Table 1). Additionally, none of the eight isolates belonged to the same NCBI SNP cluster, indicating that they were not closely related.

**TABLE 1:** *Salmonella* Dublin strain information for hydrogen peroxide killing assay

FSL ID^a^	Collection year	Host	Group
FSL R12-2657	1993	Human	NHA
FSL R12-2664	2005	Human	NHA
FSL R12-2662	2003	Cattle	NHA
FSL R12-2660	2002	Cattle	NHA
FSL R9-3257	2011	Human	HA
FSL R12-2667	2009	Human	HA
FSL R9-3231	2008	Cattle	HA
FSL R8-7251	2010	Cattle	HA

^a^ Food Safety Laboratory (FSL) identification numbers were retrieved from Food Microbe Tracker (https://www.foodmicrobetracker.net).

### Hydrogen peroxide killing assay

To determine the hydrogen peroxide concentration and exposure time for isolates, an initial hydrogen peroxide killing assay was conducted using four isolates (two NHA and two HA). The assay was conducted according to Bang et al. (34) with modifications. Briefly, all isolates’ stocks were maintained in Luria-Bertani (LB-Lennox; Difco, Detroit, MI) broth with 5 g/L NaCl and 15% (vol/vol) glycerol (brand) at −80°C. The stock cultures were streaked on LB agar, followed by overnight incubation at 37°C. Single colonies obtained on these plates were inoculated into 5 mL of LB broth, followed by incubation at 37°C with shaking at 200 rpm for 18 h. The resulting bacterial cultures were subcultured (1:1,000) into 5 mL of LB broth, followed by incubation at 37°C with shaking at 200 rpm until they reached mid-log phase (OD_600_ of 0.3–0.4; 1–2 × 10^8^ colony forming unit [CFU]/mL). Upon reaching mid-log phase, each culture was diluted 1:100 in 10 mL of LB broth to reach a cell density of approximately 1–2 × 10^6^ CFU/mL, and aliquots were taken from the diluted culture for enumeration. Subsequently, the isolates were exposed, in triplicate, to hydrogen peroxide (Sigma-Aldrich, St. Louis, MO) at 0 mM (control) and six additional concentrations ranging from 5 to 7.5 mM, in 0.5 mM increments, followed by incubation at 37°C without shaking for 30 minutes, 1 h, and 2 h before enumeration. For enumeration, a 10-fold serial dilution was performed in a microtiter plate, and 20 µL was spot plated onto LB agar supplemented with 200 U/mL of bovine catalase (MP Biomedicals, Santa Ana, CA). The initial trial indicated that exposure to 6.5 mM of hydrogen peroxide for 1 h at 37°C (without shaking) resulted in log reductions between 0.29 and 2.84 log (data not shown). Therefore, this condition was selected for conducting the assay of all eight isolates, following the same procedure as described above, with two biological replicates. For the initial trial and each biological replicate, a new bottle of hydrogen peroxide was used due to the reagent’s loss of activity.

### Statistical analysis of hydrogen peroxide killing assay

The experimental design (i.e., testing four HA isolates and four NHA isolates) provided an 80% power (β) to detect a minimum difference of 1.0 log reduction between HA and NHA groups, assuming a variance of 0.25 log reduction^2^ at a significance level (α) of 0.05. Statistical analyses were conducted within the R Statistical Programming Environment (35). To evaluate the association of the log reduction with different strain characteristics, we used a mixed-effect linear model with strain as a random effect and host and group as fixed effects. The model was implemented using the “lmerTest” package (36) in R. Prior to fitting the model, we also performed additional tests to ensure that key assumptions, which are normality of residuals and homogeneity of variance, were met. To evaluate the normality assumption, we used the Shapiro-Wilk test (37) and examined the Quantile-Quantile (QQ) plot. The Shapiro-Wilk test provided a formal assessment of normality, with the QQ plot offering a visual representation to further validate the results. Additionally, to assess the homogeneity of variance assumption, we used Levene’s test (38), which quantitatively measures the equality of variances across the fixed effect variables (i.e., host and groups). Complementing this, a residual plot was generated to visually inspect the distribution of residuals and ensure the assumption of homogeneity of variance was adequately met. The significance threshold for all statistical tests mentioned above was established at a *P*-value of 0.05.

## RESULTS AND DISCUSSION

*S*. Dublin infections in humans are typically associated with severe symptoms resulting from systemic infection (1, 39). More specifically, it has been estimated that 64% of the human infections associated with *S*. Dublin in the USA were extra-intestinal, with a 3% fatality rate. For comparison, only 6% and 7% of the *Salmonella enterica* subsp. *enterica* serovar Typhimurium and *S*. Enteritidis infections were extra-intestinal, and their fatality rate was 0.6% and 0.5%, respectively (40). In addition to its apparent increased invasiveness, it has been reported that most *S*. Dublin in the USA are MDR (41, 42). In this study, we identified HA and NHA groups within an *S*. Dublin clade (i.e., Dublin 2-3) that included more than 66% of the *S*. Dublin isolates on NCBI Pathogen Detection (15). We used comparative genomics to identify genomic features (i.e., genes and SNPs) that could be responsible for the apparent enhanced human virulence of HA isolates within *S*. Dublin clade 2-3. Informed by the identified genomic features, a hydrogen peroxide killing assay was performed to assess whether HA isolates were more resistant than NHA isolates to oxidative stress. Our results showed that (i) the well-studied *Salmonella* virulence factor, pSV, is not associated with the HA group, which we considered to represent a group with enhanced virulence, (ii) certain SNPs leading to PMSCs in virulence-associated genes are associated with isolates belonging to either the HA or NHA groups, and (iii) while the insertion of an IS*1* transposon between two genes putatively involved in oxidative stress is highly associated with the HA group, isolates in the HA group do not differ from isolates in the NHA group in terms of their tolerance to hydrogen peroxide.

### *Salmonella* virulence plasmid (pSV) is not associated with the HA group of *S*. Dublin

The pan-genome estimated among representative isolates from the HA and NHA groups comprises 5,242 genes, of which 4,205 were classified as core genes (i.e., genes present in all 80 genome assemblies), while 1,037 were categorized as accessory genes (Fig. S1).

**Fig 1:**
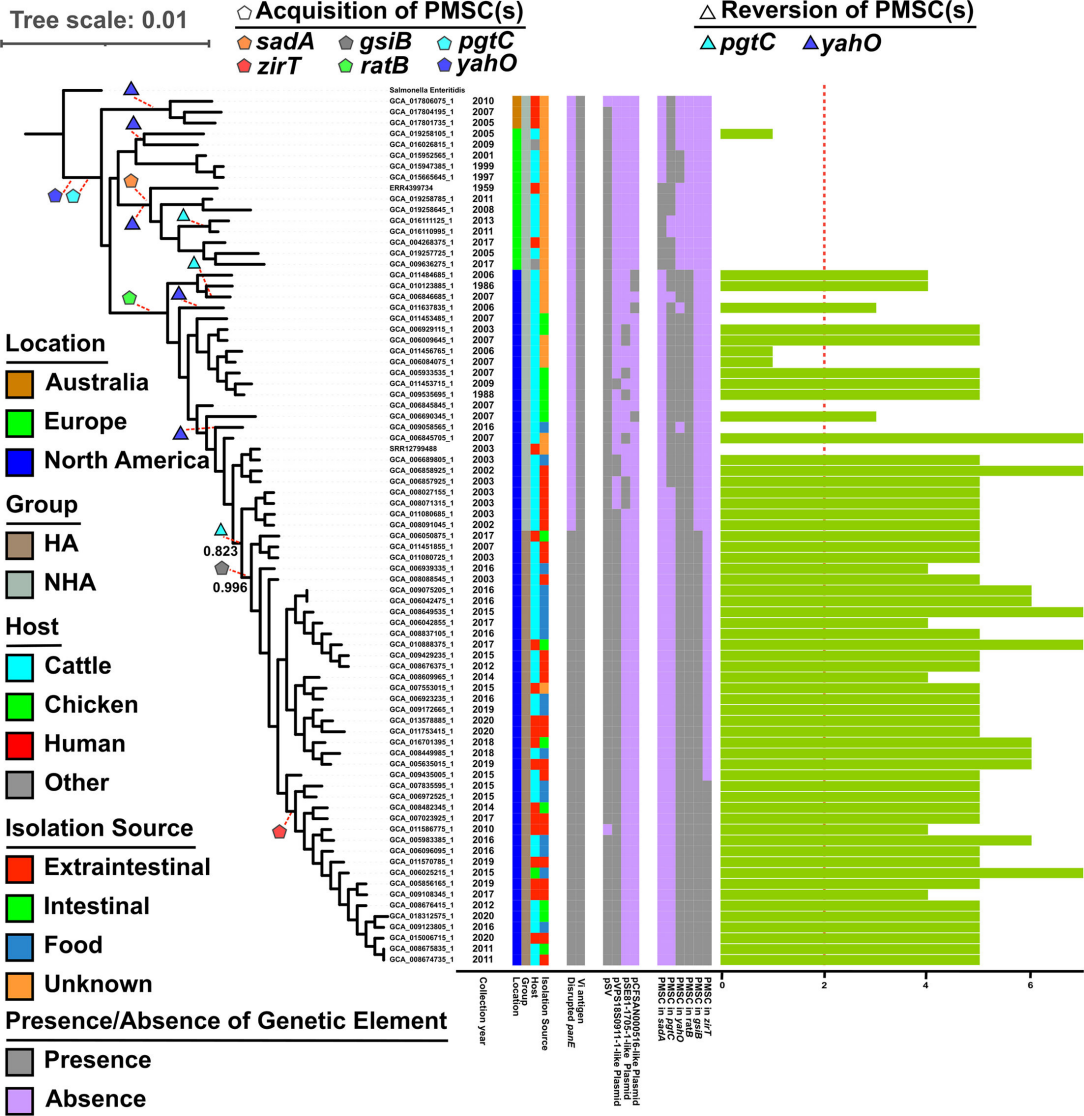
Maximum-likelihood tree of 80 *Salmonella* Dublin isolates representing the HA and NHA groups within Dublin 2-3 with gene, plasmid, PMSC, and multidrug resistance annotations. The tree was built based on an alignment of 1,695 core SNPs identified by kSNP4. Bootstrap values (based on 1,000 replicates) were denoted next to the node representing the most recent common ancestor of the HA group and the node where HA diverged from the NHA group. Location, group, host, and isolation source are color-coded. The presence of each genetic feature, including disrupted *panE* (by IS*1* transposase), Vi antigen, *Salmonella* virulence plasmid (pSV, ~74.6 kbp), pVPS18S0911-1-like plasmid (~101.8 kbp), pCFSAN000516-like plasmid (~66 kbp), and pSE81-1705-1-like plasmid (~200 kbp), is shown in gray, while its absence is indicated in purple. Pentagons and triangles represent the acquisition and reversion of PMSC(s) in the gene of interest, respectively. These events were identified by comparing the presence/absence of PMSCs among genomes relative to their most recent common ancestor, then mapped to the ancestral nodes where acquisition and reversion most likely occurred following the principle of parsimony. Each shape is connected to the ancestral node via a red dashed line to indicate where the event likely occurred. The green bar indicates the number of drug classes to which the isolate is resistant, with the red dashed line representing two drug classes. One *Salmonella* Enteritidis genome (GCF_000623455.2) is selected as the outgroup to root the tree. The scale bar at the top left reflects a distance of 0.01, representing 0.01 substitutions per site.

The *Salmonella* virulence plasmids, pSV, are 50 kb to 285 kb *Salmonella* plasmids that can be found in isolates from a small fraction of subspecies *enterica* serovars, including *S*. Dublin, *S*. Enteritidis, and *S*. Typhimurium (43). The pSVs are characterized primarily by carrying the *spv* operon, which has been shown to contribute to virulence by modulating host immune responses and intracellular infections. Interestingly, however, pSV was present in 78 of 80 isolates from both the HA and NHA groups; one human isolate in each group lacked this plasmid (Fig. 1), which may have been the result of plasmid loss during culture or may suggest that the individuals were more susceptible (e.g., immunocompromised individuals) to an infection resulting from strains not fully virulent (because of lack of the pSV plasmid). The widespread presence of pSV in both the HA and NHA groups suggests that while pSV may play a role in the virulence of *S*. Dublin, it is unlikely to be the primary factor driving the differences in association with human infections between HA and NHA groups. A previous study analyzing numerous *S*. Typhimurium clinical isolates concluded that, although pSV was present in most isolates, it might not predispose individuals to bacteremia, as its presence did not correlate with the occurrence of septicemia (43). The fact that all cattle isolates in this study carried pSV reveals that pSV may be required for virulence in cattle, which is consistent with Wallis et al.’s finding that pSV mediates the persistence of *S*. Dublin at systemic sites in cattle (44).

### An MDR plasmid is overrepresented among *S*. Dublin isolates in the HA group, while the NHA group demonstrates lower-level resistance due to the absence of that plasmid

Accessory genes contribute importantly to the genetic variability among isolates and have been suggested to enable adaptations to specific environmental conditions or specialized niches, such as increased resilience to stress conditions or the ability to exploit unique ecological opportunities (43). The HA and NHA groups showed an overrepresentation of 132 and 203 accessory genes, respectively (see Table S3 for full list). Within the HA group, 7 and 125 overrepresented genes are chromosomal and plasmid-borne, respectively, while within the NHA group, 14 and 189 overrepresented genes are chromosomal and plasmid-borne (Table S3). None of the genes overrepresented in the HA or NHA group are phage-borne. Although NHA isolates tend to have more plasmid-borne overrepresented genes, this is likely due to eight isolates from the NHA group carrying a large plasmid (pSE81-1705-1-like plasmid; ~200 kbp) not found in any of the other 72 isolates analyzed here (Fig. 1). In addition, all 40 isolates within the HA group and 5 isolates within the NHA group carried the pVPS18S0911-1-like plasmid (~101.8 kbp). The near-universal presence of pVPS18S0911-1 or its variants in the HA group suggests that the acquisition of this mobile genetic element may have coincided with the divergence of the HA lineage. Finally, four NHA isolates carried the pCFSAN000516-like plasmid (~66 kbp). Presence of pSE81-1705-1-like, pVPS18S0911-1-like, or pCFSAN000516-like plasmids was associated with MDR in isolates harboring these plasmids. The 32 isolates in the NHA group, which lacked any of these three plasmids, were identified as pan-susceptible (showing no resistance to any antimicrobials). HA isolates harbored genes conferring resistance to a significantly (*P* < 0.0001; *t*-test) higher number of AMR classes (average = 5.2 AMR classes) as compared to NHA isolates (average = 2.2 AMR classes). The AMR classes that were significantly associated with HA isolates included aminoglycoside (*P* < 0.0001), β-lactam (*P* < 0.0001), fluoroquinolone/quinolone (*P* = 0.004), phenicol (*P* < 0.0001), sulfonamide (*P* < 0.0001), and tetracycline (*P* < 0.0001). No AMR classes were associated with the NHA group.

In total, 17/40 NHA isolates and 40/40 HA isolates were considered MDR. *S*. Dublin exhibits striking geographic differences in MDR. Our results revealed that non-US isolates from Europe and Australia are predominantly pan-susceptible and lack AMR determinants, as noted by Caroll et al., who reported that the European-dominated clade within *S*. Dublin major clade I is largely pan-susceptible, while the North American group is almost exclusively MDR (45). Our findings align with this pattern, showing that almost all US Dublin isolates, especially those in the HA group, exhibit MDR phenotypes. These findings underscore the distinct MDR profiles of *S*. Dublin isolates across geographic regions and epidemiological groups, as well as the pivotal role of mobile genetic elements in shaping these resistance patterns.

There is a complex relationship between bacterial virulence and AMR. It is believed that MDR may enhance virulence to some degree, as the body cannot clear the infection quickly, resulting in excessive inflammatory responses, relapses of infections, and persistence of pathogens in the host (46, 47). Harvey et al. noted that the MDR profile of *S*. Dublin is likely to lead to prolonged hospitalization and increased clinical severity (48). Therefore, the strong association of the HA group with plasmids carrying AMR genes may help explain the potential enhanced human virulence of the HA, which could in part be attributed to prolonged infection.

### Four PMSCs disrupting genes are overrepresented in the HA group, while PMSCs in *pgtC* and *sadA* are exclusively present in isolates from the NHA group

A total of 1,695 core SNPs were identified among the 80 representative isolates. Apart from 1,037 SNPs leading to synonymous mutations and 66 SNPs leading to mutations within noncoding regions, we identified 592 SNPs leading to nonsynonymous mutations, including six nonsense mutations leading to PMSCs. As previous studies have linked PMSCs with reduced virulence in other non-typhoidal *Salmonella* serovars (49, 50), our downstream analyses were focused on these six PMSCs, comprising two overrepresented in the NHA group and four overrepresented in the HA group (Table 2).

**TABLE 2:** Genes disrupted by premature stop codons overrepresented among isolates from the NHA and HA groups

Gene	NHA/HA isolates with premature stop codons	Adjusted *P*-value^*a*^	Length of intact protein (aa)	Length of disrupted protein (aa)	% of the full-length protein	Gene product annotation
Genes with premature stop codons overrepresented among the NHA group						
*sadA*	8/0	0.040	1,342	319	23.8	Trimeric autotransporter adhesin SadA
*pgtC*	33/0	<0.001	424	166	39.2	Phosphoglycerate transport regulator PgtC
Genes with premature stop codons overrepresented among the HA group						
*zirT*	0/17	<0.001	660	622	94.2	Two-partner secretion translocator ZirT
*gsiB*	0/40	<0.001	512	476	93.0	Glutathione ABC transporter substrate-binding protein GsiB
*yahO*	25/40	<0.001	443	58	13.1	Periplasmic protein YahO
*ratB*	24/40	<0.001	2,431	11	0.5	Outer membrane protein RatB

^a^ Benjamini-Hochberg adjusted *P*-value of the Fisher’s exact tests.

PMSCs overrepresented among NHA isolates were present in (i) the gene (*sadA*) that encodes the trimeric autotransporter adhesin SadA (truncated at 23.8% of the full length) and (ii) the gene (*pgtC*) that encodes the phosphoglycerate transport regulator PgtC (truncated at 39.2% of the full length). These truncations likely render the two proteins non-functional. Notably, PMSCs were found in *sadA* and *pgtC* in 8 and 33 isolates in the NHA group, respectively, but were not found in any HA isolates (Fig. 1 and Table 2). Acquisition of PMSC in *sadA* appeared to have happened within NHA. A previous study suggested that *sadA* contributes to biofilm formation, and expression of SadA also results in increased adhesion to human intestinal Caco-2 epithelial cells (51). Therefore, the presence of eight isolates with disrupted *sadA* solely in the NHA group suggests that the accumulation of this PMSC might, at least in part, contribute to the hypovirulence of the NHA isolates due to truncation in a protein that plays an essential role in virulence (i.e., adhesion to host cells). Acquisition of PMSCs in *pgtC* appeared to have happened before the common ancestor of the 80 NHA and HA isolates. However, reversion to full-length *pgtC* appeared to have happened three times independently, two times within NHA isolates, and one time in the ancestor of all 40 HA isolates and 4 NHA isolates (Fig. 1). PgtC has been suggested to regulate the phosphoglycerate transport system and may function in conjunction with the PgtA/PgtB signaling proteins (52). No evidence has been established linking PgtC to virulence in *Salmonella*.

PMSCs overrepresented among HA isolates were present in (i) the gene (*zirT*) that encodes the two-partner secretion translocator ZirT (truncated at 94.2% of the full length), (ii) the gene (*gsiB*) that encodes the glutathione ABC transporter substrate-binding protein GsiB (truncated at 93.0% of the full length), (iii) the gene (*yahO*) that encodes the periplasmic protein YahO (truncated at 13.1% of the full length), and (iv) the gene (*ratB*) that encodes the outer membrane protein RatB (truncated at 0.5% of the full length) (Table 2). The four PMSCs that were overrepresented in the HA group included (i) two PMSCs (in *zirT* and *gsiB*) solely present in the HA isolates and (ii) two PMSCs (in *yahO* and *ratB*) found in both the HA and NHA groups. PMSCs in *zirT* and *gsiB* seem to have happened once each, taking place in the ancestor of 17 and 40 HA isolates, respectively. Gal-Mor et al. (53) observed that the ZirTS secretion pathway, mediated by *zirT* and *zirS*, acts as an antivirulence modulator during infection. These authors also suggested that, *in vivo*, *zirT* is specifically induced in *Salmonella* during the colonization of the small intestine rather than in systemic sites (e.g., bloodstream or internal organs). During both acute and persistent infections in mice, the authors observed that high levels of *zirT* expression occurred in *Salmonella* shed in feces. Hence, their results indicated that inactivation of the ZirTS pathway leads to a hypervirulent phenotype in *Salmonella* in the course of oral infection in mice, as the role of ZirTS is likely to prevent premature host death during an early stage of the infection (53). Therefore, the 17 HA isolates with disrupted *zirT* may present higher virulence compared to other isolates, consistent with the overrepresentation of human isolates in the HA group (45%) compared to the NHA group (12%). In fact, six of the nine human isolates obtained from extra-intestinal body sites (e.g., blood, spleen) harbored the *zirT* PMSC (Fig. 1). Since the PMSC is located near the end of *zirT* (i.e., 94.2% of the full length), whether the function of the truncated ZirT is maintained requires further investigation. Another disrupted gene in HA isolates, *gsiB*, is predicted to encode the glutathione-binding protein GsiB, which is involved in the glutathione uptake system (54). It is unknown whether *gsiB* is associated with virulence in *Salmonella*. Notably, the PMSC in *gsiB* was ubiquitous in the HA group, as all HA isolates (*n* = 40) exhibited this PMSC. Conversely, all NHA isolates (*n* = 40) possessed an intact *gsiB*. However, due to the position of the PMSC (i.e. 93% full length), it is unclear whether the function of GsiB is affected by this PMSC.

The remaining PMSCs overrepresented in the HA group (in *yahO* and *ratB*) were found in all 40 isolates in the HA group, but also in 25 and 24 out of 40 NHA isolates, respectively. Acquisition of PMSCs in *yahO* appeared to have happened before the common ancestor of the 80 NHA and HA isolates. However, reversion to full-length *yahO* appeared to have happened five times independently, all of which were within NHA isolates (Fig. 1). Eletsky et al. (55) suggested that the periplasmic protein YahO does not appear to be related to pathogenesis. Instead, it likely functions similarly to YcfR/BhsA, YbiM/McbA, YjfO/BsmA, or YcfR, proteins with uncharacterized roles in biofilm formation and stress responses. Acquisition of PMSC in *ratB* appeared to have happened once in the ancestor of all HA isolates and 24 of the 40 NHA isolates (Fig. 1). Kingsley et al. (56) found that a *S*. Typhimurium strain with a deletion in *ratB* showed a colonization defect specifically in the cecum, but not in the spleen, Peyer’s patches, and mesenteric lymph nodes. Altogether, the PMSCs in *yahO* and *ratB* do not seem to compromise the extra-intestinal capabilities of the isolates carrying these variants.

### *panE* disrupted by an IS*1* transposon is overrepresented and only found among isolates in the HA group

**Fig 2:**
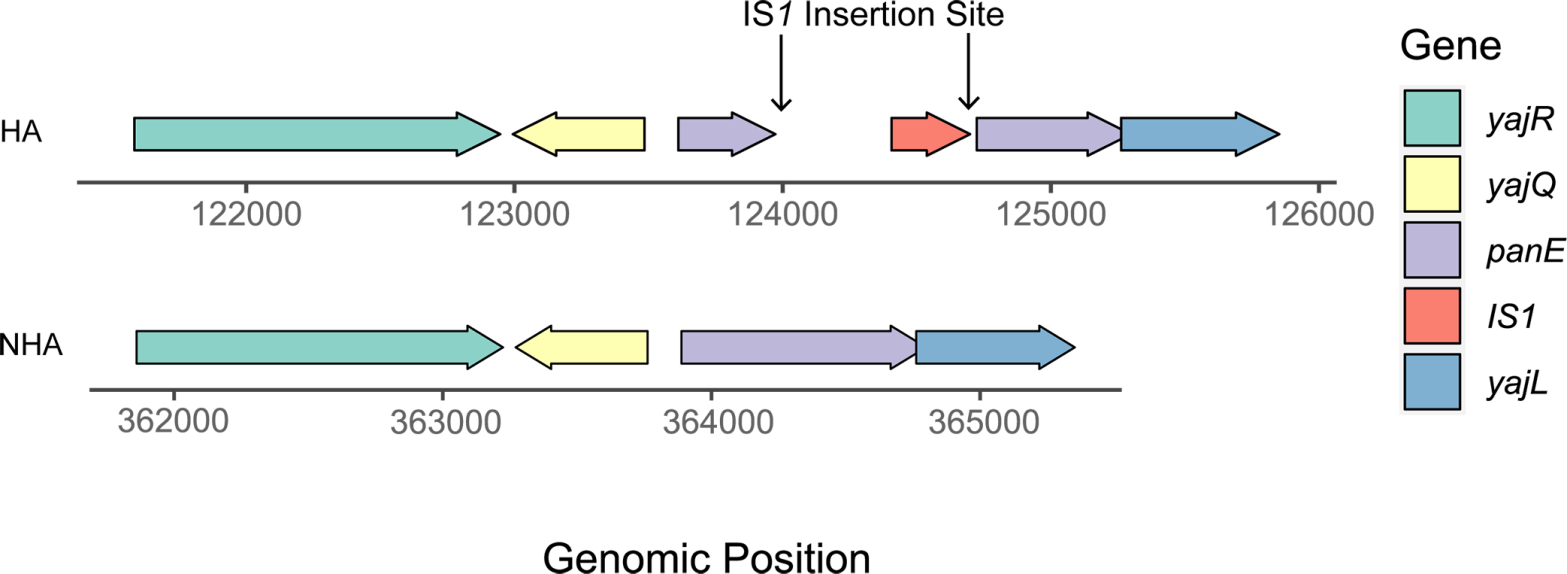
Schematic diagram of the exclusive genotypic difference between HA and NHA groups in the region between *yajR* and *yajL*. Genes are color-coded, and the direction of transcription of each gene is indicated by the arrow. The coordinates for corresponding genes are labeled on the axis, drawn to scale. An IS*1* insertion *panE* is observed in all HA sequences; this insertion is absent in all NHA sequences.

All 40 isolates in the HA group carry an IS*1* transposon inserted into *panE*, while all 40 isolates in the NHA group carry an intact copy of *panE* (Fig. 1). *panE* encodes a 2-dehydropantoate 2-reductase that converts 2-dehydropantoate to pantoate, a key precursor for the synthesis of coenzyme A (57). Although the disruption in *panE* has been found exclusively in the HA group, previous studies revealed that in both *Escherichia* (*E*.) coli and *S*. Typhimurium, a *panE* knock-out mutant by itself does not exhibit auxotrophy for pantothenate, indicating that these mutant strains retain their ability to synthesize this substrate. It has been shown that the conversion of 2-dehydropantoate to pantoate can also be carried out by a ketol-acid reductoisomerase, which is encoded by *ilvC* (58–60); all 80 isolates analyzed here carry a full copy of *ilvC*, suggesting that all isolates can synthesize pantothenate. Two genes upstream of *panE* (i.e., *yajR* and *yajQ*) are transcribed in opposite directions from each other (Fig. 2). *yajR* encodes a transporter of the major facilitator superfamily (61), suggested to be an efflux pump (62), while *yajQ* encodes the c‐di‐GMP receptor protein, which has been suggested to regulate the virulence of the plant pathogen *Xanthomonas campestris* (63, 64). Downstream of *panE* is *yajL*, with both *panE* and *yajL* being transcribed in the same direction. *yajL* encodes a protein/nucleic acid deglycase 3, which functions as a chaperone for sulfenylated proteins and has been suggested to provide protection for bacteria in response to oxidative stress and protein aggregation induced by oxidative stress (65, 66).

Previous studies have suggested that IS element insertions could be involved in the virulence of several pathogens. A previous study suggested that the virulence of *Staphylococcus aureus* is mediated by IS*256*, which inserts into the *rot* (repressor of toxins) promoter, decreasing the expression of Rot, thus driving the derepression of cytotoxin genes by Rot, leading to cytotoxin expression and increased virulence (67). Another study described that IS*Rpe1* (IS*481* family) transposition in *Rickettsia peacockii* leads to regulatory disruption of two genes associated with actin-tail polymerization and cell adhesion, therefore modulating the virulence of this pathogen (67). In *E. coli*, IS*2* has been found to be responsible for the constitutive expression of *Gal*- alleles downstream from its insertion (68). Therefore, insertion elements can affect the expression of surrounding genes in many ways, either directly (e.g., by disrupting the gene) or indirectly (e.g., by inserting new promoters upstream of surrounding genes). Upstream of the disrupted *panE*, *yajQ* expresses a putative cytoplasmic protein that has not been well characterized in *Salmonella* (69). However, Chen et al. suggested that overexpression of *yajQ* in *E. coli* causes the minimum inhibitory concentration of hydrogen peroxide (H_2_O_2_), a reactive oxygen species, to increase by more than threefold (70). The release of H_2_O_2_ is induced by macrophages while engulfing and digesting pathogens in order to clear infection (71). In response, one of *Salmonella*’s defense mechanisms involves coping with oxidative stress via resistance to reactive oxygen species (72). Downstream of the disrupted *panE*, *yajL* encodes a covalent chaperone for sulfenylated proteins that has been suggested to have antioxidative properties (65, 66). Crucially, *yajL* forms an operon with *panE* (73), resulting in the joint transcription of these two genes. Another study suggested that overexpression of YajL in *E. coli* results in increased resistance to glyoxals (74). Glyoxals, one of the reactive oxygen species, are mainly formed by lipid peroxidation (75). At concentrations of 20–100 µg/plate, glyoxals induce frameshift mutations specifically at G:C base pairs in *S*. Typhimurium (76). As an increase in intracellular survival is crucial to establish systemic infection in humans (77), we hypothesized that the inserted IS*1* transposon in *panE* could affect the expression of the putative oxidative stress response proteins YajQ or YajL in isolates from the HA group.

### The IS*1* transposon within *panE* does not promote resistance to hydrogen peroxide

**Fig 3:**
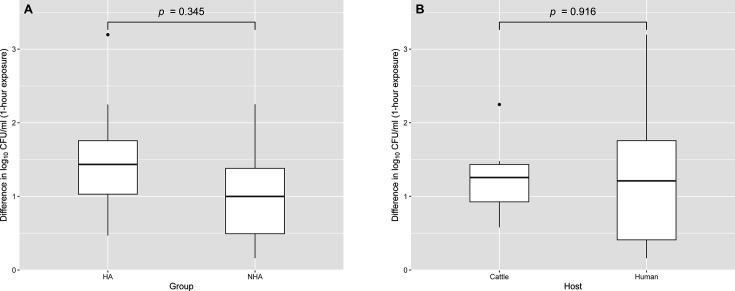
Box plots showing the log reduction of *Salmonella* Dublin across group and host after 1 h of exposure to 6.5 mM of hydrogen peroxide. Outliers are indicated as individual points. A total of eight isolates were used in this experiment, divided into two categories: between groups (HA, *n* = 4; NHA, *n* = 4) and between hosts (cattle, *n* = 4; human, *n* = 4). (**A**) shows the comparison between HA and NHA groups, while (**B**) shows the comparison between isolates of cattle and human origin. Each isolate was tested in two biological replicates, with each replicate run in technical triplicates. Statistical analysis was performed using a mixed-effect linear model with strain as a random effect and host and group as fixed effects.

The hydrogen peroxide killing assay, conducted at a concentration of 6.5 mM, revealed no significant difference in resistance to hydrogen peroxide between NHA and HA strains (Fig. 3A). The log reduction (CFU/mL) varied from 0.20 to 1.61 for NHA strains and from 0.96 to 2.40 for HA strains (Table S4). The mean log reductions in the NHA and HA groups were 1.00 and 1.52, respectively (Table S4). While the HA strains showed slightly higher log reductions compared to the NHA strains, this difference was not statistically significant. A mixed-effect linear model showed no significant difference between the two groups (*P* = 0.345).

Statistical analysis also showed no significant effect (*P* = 0.916) of the IS*1* transposon in the survival to H_2_O_2_ in strains isolated from different hosts, including humans and cattle (Fig. 3B). In the human host group, log reductions ranged from 0.20 to 2.40, with a mean of 1.29, while in the cattle host group, log reductions ranged from 0.96 to 1.71, with a mean of 1.23, as shown in Table S4.

Our findings suggest that the IS*1* transposon insertion between two genes involved in oxidative stress does not interfere in oxidative stress resistance, specifically resistance to hydrogen peroxide, in *S*. Dublin. Hydrogen peroxide is a key antimicrobial defense mechanism employed by host immune cells, particularly neutrophils and macrophages, to control bacterial infections (78). Although the IS*1* transposon within *panE* does not appear to interfere with the resistance to H_₂_O_₂_ in *S*. Dublin strains under the experimental conditions tested, the non-significant results of the H_2_O_2_ killing assay do not necessarily indicate that the disruption by the IS*1* transposon is irrelevant to virulence. Assays using animal model experiments to assess systemic virulence may, in the future, show a significant effect of the IS*1* insertion in oxidative survival or virulence of isolates carrying this insertion. *Salmonella* is equipped with multiple systems to cope with oxidative stress (72). The H_2_O_2_ killing assay used in this study may not capture the full range of these mechanisms. While this study aimed to evaluate whether HA and NHA isolates exhibit differential tolerance to oxidative stress using H_2_O_2_—a widely utilized and time-efficient oxidative agent—our findings revealed no significant differences between the two groups. However, it remains possible that HA and NHA isolates may respond differently to other reactive oxygen species, such as glyoxals. Further research is needed to explore this possibility.

## SUPPLEMENTAL MATERIAL

Fig. S1 spectrum.01336-25-s0001.docx Pie chart of the pangenome composition of Dublin 2-3.

Table S1 spectrum.01336-25-s0002.xlsx Metadata for 80 genomes used in this study.

Table S2 spectrum.01336-25-s0003.xlsx Reference genomes used in the SNP analysis for identifying premature stop codons.

Table S3 spectrum.01336-25-s0004.xlsx Full list of overrepresented genes among isolates from HA and NHA groups.

Table S4 spectrum.01336-25-s0005.xlsx Hydrogen peroxide killing assay enumeration results for groups and hosts.

ASM does not own the copyrights to Supplemental Material that may be linked to, or accessed through, an article. The authors have granted ASM a non-exclusive, world-wide license to publish the Supplemental Material files. Please contact the corresponding author directly for reuse.
