# Supplementary material for: Novel genetic features associated with the recently emerged MDR clade of Salmonella Dublin linked to human clinical cases
Source: Microbiol Spectr. 2025 Jul 18;13(9):e01336-25. doi: 10.1128/spectrum.01336-25 (PMC12403712; doi:10.1128/spectrum.01336-25)
Supplement: Fig. S1 — Pie chart of the pangenome composition of Dublin 2-3. [file spectrum.01336-25-s0001.docx]

**
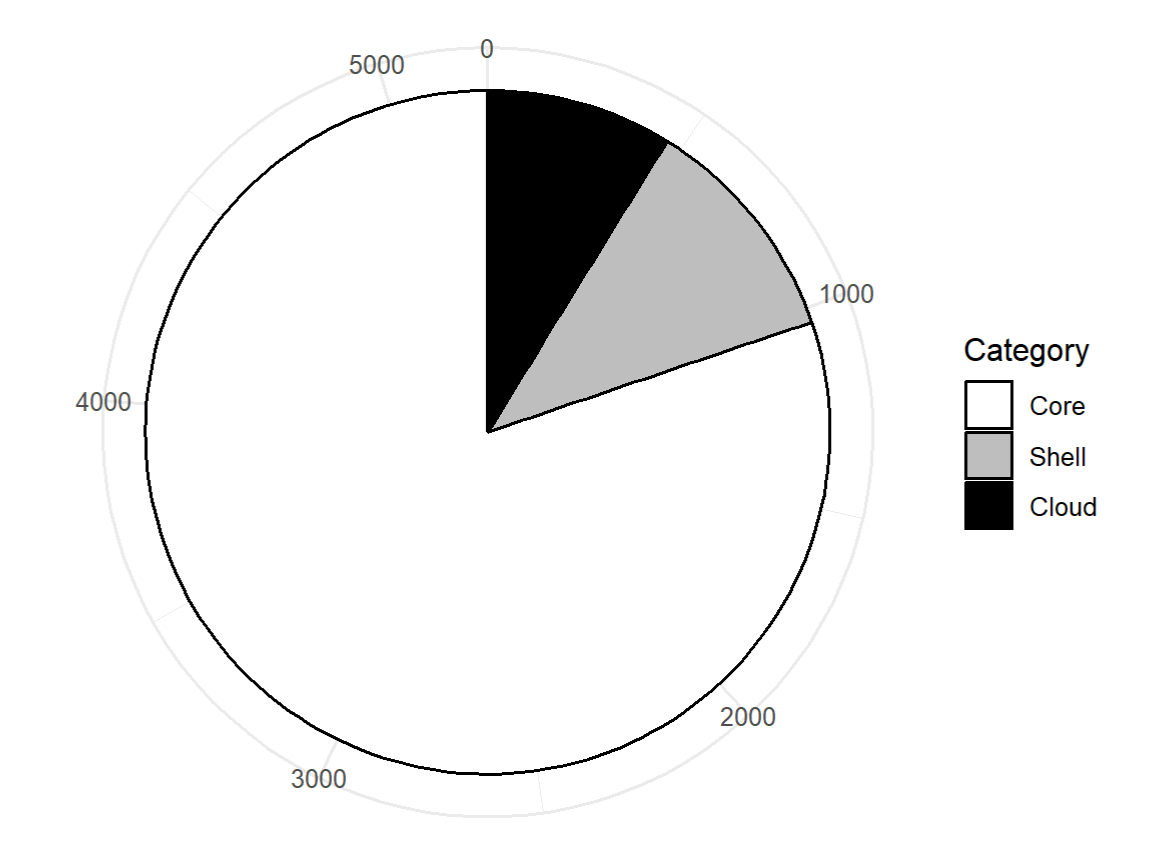
**

**Supplementary Figure 1.** Pie chart of the pangenome composition of Dublin 2-3. Core genes are found in every isolate (100%) in this study, shell genes are present in 15–99% of isolates, and cloud genes are found in less than 15% of isolates.
